# The differential contribution of tumour necrosis factor to thermal and mechanical hyperalgesia during chronic inflammation

**DOI:** 10.1186/ar1743

**Published:** 2005-04-12

**Authors:** Julia J Inglis, Ahuva Nissim, Delphine M Lees, Stephen P Hunt, Yuti Chernajovsky, Bruce L Kidd

**Affiliations:** 1 Bone and Joint Research Unit, Barts and The London School of Medicine and Dentistry, John Vane Science Centre, London, UK; 2 Experimental Therapeutics, Barts and The London School of Medicine and Dentistry, John Vane Science Centre, London, UK; 3 Department of Anatomy and Developmental Biology, University College London, London, UK

## Abstract

Therapies directed against tumour necrosis factor (TNF) are effective for the treatment of rheumatoid arthritis and reduce pain scores in this condition. In this study, we sought to explore mechanisms by which TNF contributes to inflammatory pain in an experimental model of arthritis. The effects of an anti-TNF agent, etanercept, on behavioural pain responses arising from rat monoarthritis induced by complete Freund's adjuvant were assessed and compared with expression of TNF receptors (TNFRs) by dorsal root ganglion (DRG) cells at corresponding time points. Etanercept had no effect on evoked pain responses in normal animals but exerted a differential effect on the thermal and mechanical hyperalgesia associated with rat arthritis induced by complete Freund's adjuvant (CFA). Joint inflammation was associated with increased TNFR1 and TNFR2 expression on DRG cells, which was maintained throughout the time course of the model. TNFR1 expression was increased in neuronal cells of the DRG bilaterally after arthritis induction. In contrast, TNFR2 expression occurred exclusively on non-neuronal cells of the macrophage–monocyte lineage, with cell numbers increasing in a TNF-dependent fashion during CFA-induced arthritis. A strong correlation was observed between numbers of macrophages and the development of mechanical hyperalgesia in CFA-induced arthritis. These results highlight the potential for TNF to play a vital role in inflammatory hyperalgesia, both by a direct action on neurons via TNFR1 and by facilitating the accumulation of macrophages in the DRG via a TNFR2-mediated pathway.

## Introduction

Pain and disability are the principal clinical features associated with chronic arthritis. Inflammation is associated with sensitisation of specialised sensory neurons that comprise the nociceptive (pain) pathway, leading to enhanced pain sensations in response to both noxious and non-noxious stimuli (termed hyperalgesia and allodynia, respectively) [1]. Neural sensitisation is thought to arise in response to the actions of mediators on both peripheral and central nociceptive neurons. Whereas acute inflammatory mediators such as prostaglandins and bradykinin have been shown to exert important effects on neuronal sensitisation in the short term, the longer-term influences of these and other mediators on nociceptive neurons remain less clear.

Therapies directed against tumour necrosis factor (TNF) have proved highly effective in treating rheumatoid arthritis. In addition to exerting an anti-inflammatory effect and slowing the progression of rheumatoid arthritis, anti-TNF therapy produces a profound and rapid analgesia [2]. The mechanism of this latter action remains uncertain, but the action suggests an important role for TNF in persistent inflammatory (neuroplastic) pain.

In acute situations, TNF has been reported to sensitize nociceptive neurons indirectly via the induction of a proinflammatory cytokine cascade involving IL-1β, IL-6, and IL-8, resulting in the release of prostaglandins and other mediators from immune cells [3-5]. Evidence for more direct TNF actions comes from electrophysiological studies showing that low-dose subcutaneous injections of TNF-α induce ectopic activity in nociceptive neurons within 2 minutes, with higher doses producing significant mechanical and thermal hyperalgesia by 15 minutes [6-8]. Furthermore, application of TNF-α enhances calcium currents and increases neuron sensitivity to the neurotoxin capsaicin in cultures of sensory neurons [9-11].

TNF acts via two receptors, including the p55 TNF receptor type 1 (TNFR1) and the p75 TNF receptor 2 (TNFR2). Both receptors have been reported as being present within the rat dorsal root ganglion (DRG), although the cellular distribution remains controversial [11-13]. Recently, increased neuronal TNFR1 expression was reported in association with intraperitoneal lipopolysaccharide [13], suggesting a direct TNF effect on nociceptive pathways via TNFR1. Neuronal expression of TNFR2 has been reported by some investigators [12,14,15] but not others [13]. While these studies have been performed in normal animals or within hours of the onset of inflammation, the situation during more chronic phases of inflammatory disease remains unclear.

The antihyperalgesic actions of an anti-TNF drug, etanercept, have been investigated in an experimental neuropathic model [16], but there are no reports of the antihyperalgesic effects of these drugs in inflammatory models. Nociceptive mechanisms in neuropathic conditions differ from those present in inflammatory disorders. Therefore, our aim was to assess the effects of etanercept on various behavioural pain measures in an experimental model of persistent arthritis and to compare these with the sequential expression and cellular distribution of TNF receptors by DRG cells, particularly during the later and clinically more relevant phases of inflammatory disease.

## Materials and methods

### Animals

Adult (180 to 250 g) male Wistar Rats were kept in groups of between 3 and 5 animals in cages maintained at 20°C with a 12-hour light/dark cycle and food and water *ad libitum*. All experimental procedures were approved by the UK Home Office and followed guidelines issued by the International Association for the Study of Pain. Inflammation was induced by a single intraplantar injection (100 μl) of complete Freund's adjuvant (CFA) (Becton Dickinson, Franklin Lakes, NJ, USA) into the right hind footpad of each animal, prepared as a 10-mg/ml suspension of heat-attenuated *Mycobacterium tuberculosis *in sterile paraffin oil (Sigma, St Lois, MO, USA). For subsequent *in situ *hybridisation and real-time PCR analysis, animals were killed with CO_2_, and for immunohistochemical analysis they were killed with an overdose of phenobarbitone, followed by intracardiac perfusion with 250 ml saline and 500 ml periodate–lysine–paraformaldehyde fixative [17]. L4/L5 DRG tissues were removed for assessment.

### Drugs

Etanercept (Wyeth Pharmaceuticals, Madison, WI, USA) (0.5 mg/kg reconstituted in 0.5 ml sterile water) was administered subcutaneously on alternate days starting either 1 day before or 3 days after the induction of inflammation with CFA. Control animals received denatured etanercept that had previously been heated to 95°C for 10 min.

### Behavioural assessments

Footpad diameter was assessed with calipers before and 3 and 7 days after CFA injection (*n *= 4 each time). Behavioural pain measures were assessed at the same time points. Nociceptive thresholds to mechanical stimulation were determined using von Frey hairs of increasing gauge (0.6 to 12.6 g), as previously described [18]. Animals were placed in boxes of wire mesh and von Frey hairs were applied to the plantar surface of the hind paw. The lowest-weight von Frey hair to evoke a withdrawal from three consecutive applications was considered to indicate the threshold. Thermal hyperalgesia was assessed with the Hargreaves algometer test [19]. Animals were placed in a Perspex (methyl methacrylate) box and an increasing thermal stimulus was delivered to the plantar surface of the hind paw. The time interval to lifting of the paw was recorded. All assessments were performed with the assessor blinded with respect to treatment and were repeated on at least two separate occasions.

### *In situ *mRNA hybridisation

Primers were designed against fragments of the rat TNFR1 and TNFR2 cDNA [20,21] (Invitrogen, San Diego, CA, USA). TNFR1 (5'-CGG AAT TCC AAA GAG GTG GAG GGT GAA GGA-3' (bp 999 to 1020), 5'-CCA TCG ATC AGT GTC AAG CCG TTG TTG CTG-3' (bp 1297 to 1318)); TNFR2 (5'-CGG AAT TCC CCA GGA TGC AGT AGG CCT TGA-3' (bp 11 to 32), 5'-CCA TCG ATC AGA CGT TCA CGA TGC AGG TGA-3' (bp 230 to 253)). 320- and 242-bp fragments of the TNFR1 and TNFR2 receptors from rat brain and monocyte cDNA, respectively (gifts from Adrian Bristow, National Institute for Biological Standards and Control, UK) were cloned into PCR2.1, using a TA cloning kit (Invitrogen). Plasmids were linearised and were transcribed with DIG-UTP RNA mix (Roche, Basel, Switzerland).

*In situ *mRNA hybridisation was performed on 10 μM sections of DRG from naive rats 3 and 7 days after the induction of inflammation with CFA [22]. Labelling was detected with sheep anti-DIG-AP antibody (Roche), and NBT/BCIP (Roche).

### Real-time RT-PCR

Forward and reverse primers were designed for the TNFR1 and TNFR2 mRNA using Primer Express software (Applied Biosystems, Foster City, CA, USA). The primers chosen for TNFR1 and TNFR2 (TNFR1: 5'-CTC TTG GTG ACC GGG AGA AG-3' (bp 98-117), 5'-GGT TCC TTT GTG GCA CTT GGT-3' (bp 203-183); and TNFR2: 5'-CAT CCC TGT GTC CTT GGG-3' (bp 709–808), 5'-CCC GTG ATG CTT GGT TCA-3' (bp 839-820)) gave products of 101 and 51 bp, respectively.

Total RNA was extracted from frozen L4/L5 DRGs using Qiagen RNeasy mini-kit with on-column DNase digestion (Qiagen, Hilden, Germany), and reverse transcription was performed using a Promega Reverse Transcription kit according to the manufacturer's instructions (Promega, Madison, WI, USA). Real-time PCR was carried out with SYBR Green PCR mastermix (Applied Biosystems) containing AmpliTaq Gold [23]. Taqman real-time PCR was performed on 10 ng of each sample and the standard curve (5 to 20 ng) for 18S RNA [23] (Applied Biosystems) in a 20-μl reaction volume. The TNFR mRNA level was expressed as a ratio to 18S RNA.

For cloning and expression of the rat TNFR2, total RNA was isolated from rat spleen using a Qiagen RNeasy mini-kit with on-column DNAse digestion (Qiagen). RNA was reverse-transcribed using Promega Reverse Transcription kit (Promega). Primers were generated to amplify the full coding region of the receptor, according to the corresponding cDNA sequence in the mouse [24] (Invitrogen) (5'-CTG GGT ACC ACC ATG GCG CCC GCC GCC CTC-3', 5'-GGC CAC TTT GAC TGC AAT CT-3'). The 1384-bp fragment was cloned into PCDNA6 His B (Invitrogen). TNFR2-PCDNA6, or empty vector, was transfected into human embryonic kidney 293T cells using a CaCl_2_–glycerol shock protocol described previously [25]. The cloned receptor was sequenced and submitted to GenBank (17-07-2003, NCBI Accession number AY344841).

### Production and characterisation of an anti-TNFR2 single-chain variable fragment (ScFv)

Selection was carried out as described by White and colleagues [26], with minor modifications. 293T cells were seeded in 6-well plates at 5 × 10^5^. One well per plate was transfected with TNFR2, while two were left untransfected. Forty-eight hours after transfection, 500 μl Tomlinson J-library phage [27], prepared as described at , (2 × 10^11^TU) was depleted on untransfected 293T cells, and unbound phage were collected. Unbound phage was then applied to a transfected culture and bound phage was eluted by incubation with 500 μl 100 mM triethylamine, pH 12. Three rounds of selection were performed. After the final round, phage was infected into HB2151 bacteria, and monoclonal ScFv ELISA was performed against TNFR2-transfected cell lysate and vector-transfected lysate. A strongly binding clone was produced.

To ensure specificity, double immunohistochemistry was performed on TNFR2-transfected 293T cells, first with anti-His FITC (Qiagen) in accordance with the manufacturer's instructions. Subsequently, cells were stained with ScFv and detected using anti-myc-CY-3 antibody (Sigma, St Louis, MO, USA). To further assess specificity, immunoprecipitation was performed against 293T cells expressing His-tagged TNFR1, or TNFR2 using the produced ScFv, and an anti-His antibody (Qiagen). ScFv or mouse anti-His antibody (5 μg) was mixed with 20 μl 50% (w/v) Protein A beads (Amersham, Little Chalfont, UK) and 100 μg TNFR1- or TNFR2-transfected 293T lysate. Protease cocktail inhibitors were added, and solutions were incubated overnight with agitation at 4°C. Protein A beads were centrifuged and washed three times with 1 ml PBS to remove non-bound protein. Beads were then resuspended in 30 μl SDS loading dye and heated for 10 min at 95°C. Samples were then run on a 10% SDS gel and probed with an anti-His antibody.

### Immunohistochemistry and histology

L4/L5 DRGs were removed and cryoprotected overnight in a 30% sucrose solution. 10 μM sections of the DRGs were stained with mouse anti-ED1 (Serotec, Oxford, UK), rabbit anti-ATF-3 (Santa Cruz, Santa Cruz, CA, USA), rabbit anti-GFAP (Dako Cytomation, Glostrup, Denmark), guinea pig anti VR-1 (Chemicon, Temecula, CA, USA), or human antirat TNFR2 ScFv. Detection was performed with fluorescently labelled conjugates (Jackson Immunolabs, West Grove, PA, USA). An anti-His Alexa Fluor 488 (Qiagen) was used for detection of ScFv binding. ED-1-positive cells adjacent to neuronal somata were counted, and the results were expressed as the ratio of the number of positive cells to the number of neuronal somata [28]. Macrophages with a nucleus were counted; hence measurements are conservative. Joints were decalcified, embedded in paraffin wax, sectioned, and stained with haematoxylin and eosin. Joints were assessed for inflammatory infiltrate, bone necrosis, and cartilage degradation [17]. Joints were scored from 0, indicating no damage, to 4, indicating severe damage or infiltration. Assessments were performed in a blinded fashion by a trained investigator (BLK).

### Data analysis

Statistical analysis was performed using one-way analysis of variance with post hoc Bonferroni multiple range testing and unpaired *t*-test for comparing two means. Linear regression and Pearson's correlation were performed to assess the relation between macrophage numbers and mechanical hyperalgesia.

## Results

### Characteristics of CFA-induced arthritis

**Figure 1:**
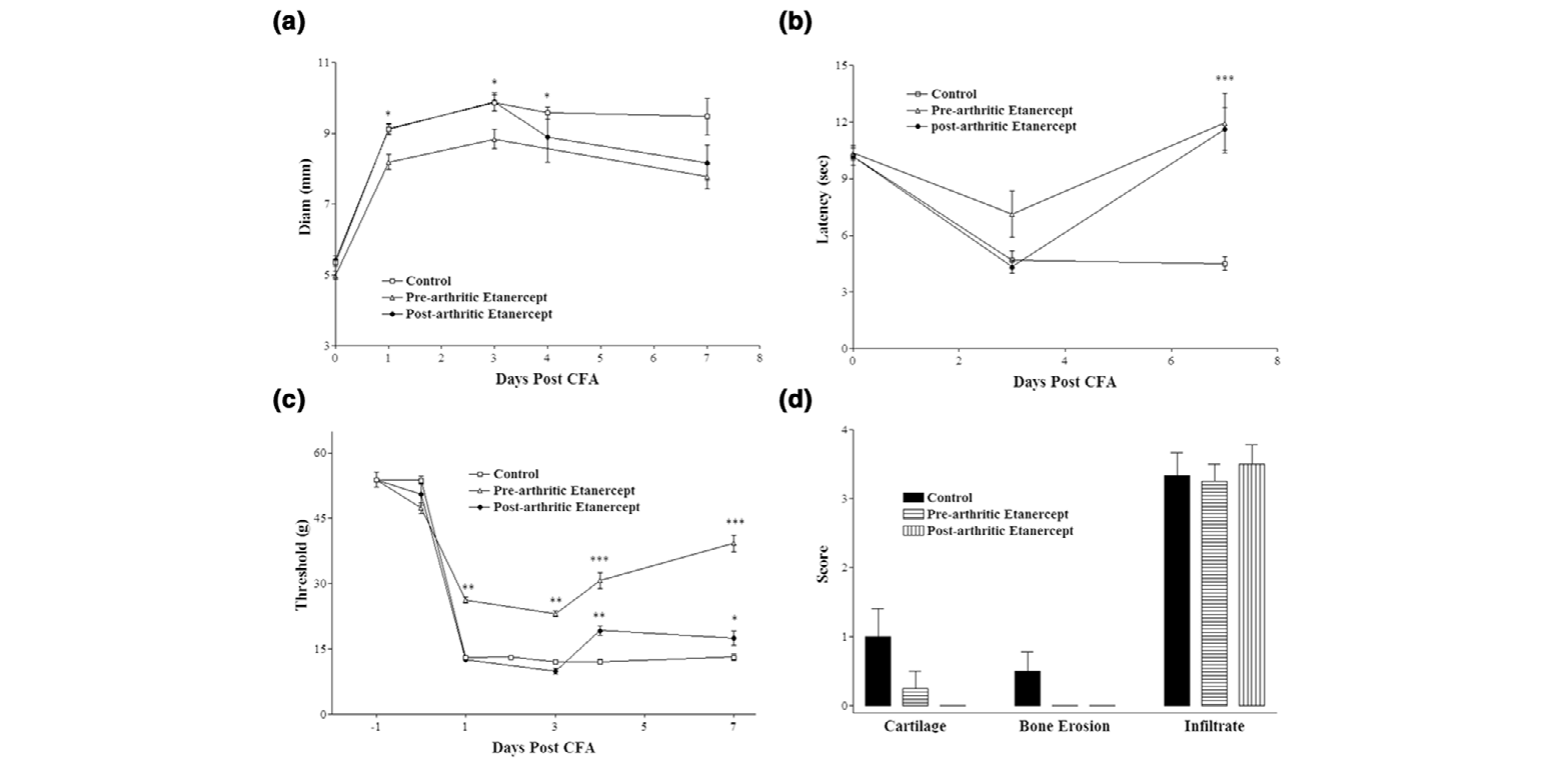
Etanercept treatment attenuates mechanical and thermal hyperalgesia, with little effect on swelling and histological damage. **(a) **Prearthritic etanercept treatment in rats with arthritis induced by complete Freund's adjuvant (CFA) reduced paw swelling significantly, by 10%, until 4 days after inflammation induction (*n *= 4). Thermal **(b) **and mechanical **(c) **hyperalgesia were assessed throughout the study. (b)Both prearthritic and postarthritic etanercept therapy abolished thermal hyperalgesia 7 days after inflammation (*n *= 4). (c) Prearthritic etanercept treatment reduced mechanical hyperalgesia significantly throughout the period studied, and postarthritic treatment reduced hyperalgesia to a lesser extent. **(d) **Joints were stained with haematoxylin and eosin and were assessed on a 4-point scale (where 0 = absent, 1 = mild, 2 = moderate, 3 = severe) for severity of inflammatory infiltrate, bone necrosis, and cartilage damage. CFA induced inflammatory infiltration 3 days (not shown) and 7 days (d) after CFA, while bone and cartilage were largely preserved. We found no reduction in histological score with either etanercept treatment regime. *P < 0.05; ***P < 0.005 in comparison with controls.

Administration of CFA produced a twofold increase in paw diameter in control arthritic animals treated with denatured etanercept (Fig. 1a) as well as a reduction in both the thermal withdrawal latency (Fig. 1b) and the mechanical withdrawal threshold (Fig. 1c). Histological assessment demonstrated a pronounced inflammatory infiltrate with modest bone necrosis and cartilage loss (Fig. 1d).

### Effects of TNF antagonism on CFA-induced arthritis

The effects of the TNF antagonist etanercept on inflammation and behavioural pain measurements during CFA arthritis were assessed. Etanercept produced a modest decrease in paw swelling when given either before or after the onset of inflammation (Fig. 1a). No gross histological differences were seen between etanercept-treated and control-treated animals (Fig. 1d).

Treatment with etanercept before the onset of inflammation had no effect on thermal withdrawal latencies in naive animals or those tested 3 days after CFA injection, but abolished thermal hyperalgesia at 7 days (Fig. 1b). It was notable that treatment with etanercept after the onset of inflammation also abolished thermal hyperalgesia at the 7-day time point.

Mechanical hyperalgesia in naive animals was unaffected by etanercept. When administered before the onset of inflammation, etanercept significantly attenuated mechanical hyperalgesia such that the withdrawal threshold increased from 12 g to 23 g at 3 days and to 39 g at 7 days (*P *< 0.05). In contrast, little effect was observed on mechanical hyperalgesia when etanercept treatment was commenced 3 days after injection of CFA (Fig. 1c).

### TNF receptor mRNA expression in rat dorsal root ganglia during CFA arthritis

**Figure 2:**
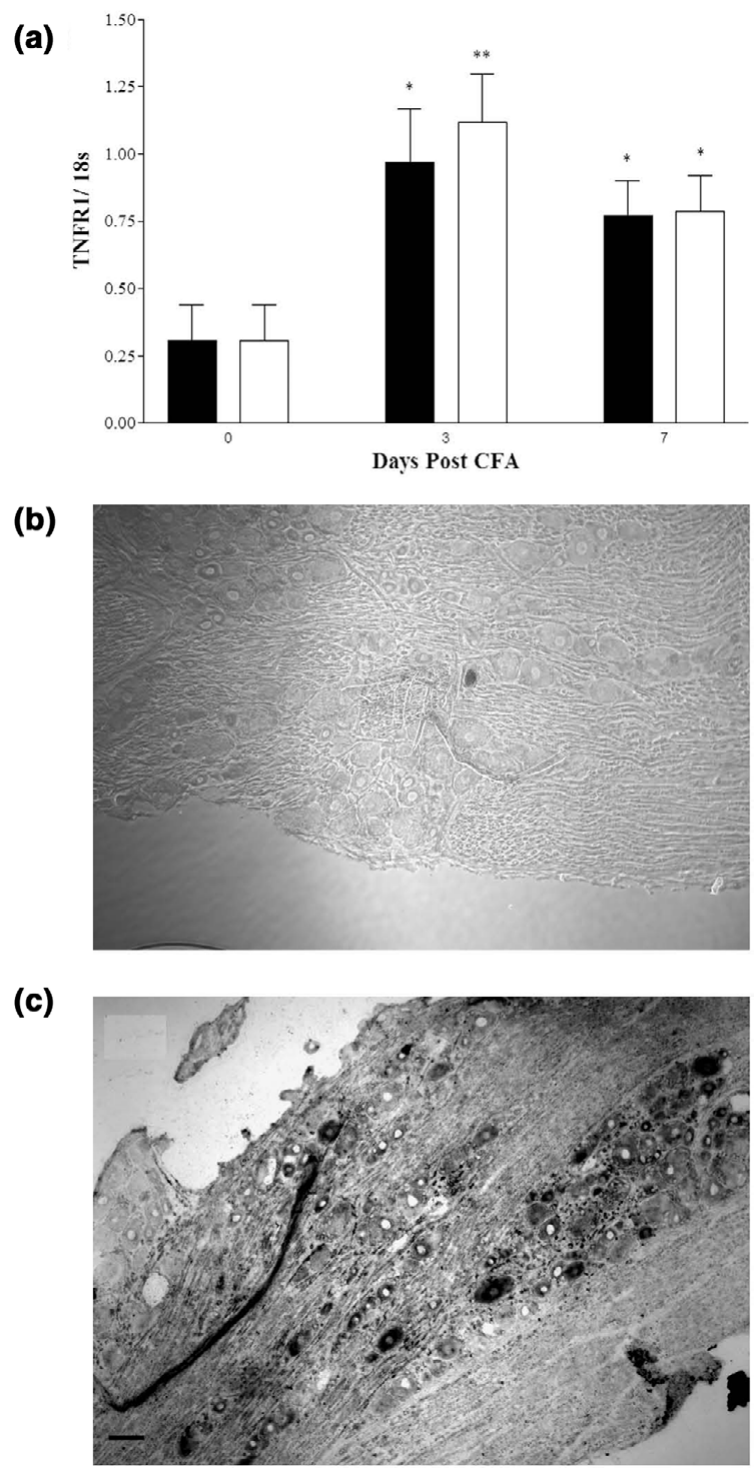
TNFR1 mRNA is increased following inflammation in the dorsal root ganglion (DRG). **(a) **Real-time RT-PCR showed a threefold increase in mRNA of tumour necrosis receptor type 1 (TNFR1) (expressed as the ratio of TNFR1 mRNA to 18S mRNA) in the DRG following the induction of inflammation by complete Freund's adjuvant (CFA) (*n *= 4). The increase was bilateral, being observed on both the ipsilateral (black bars) and the contralateral (white bars) DRGs. **(b) ***In situ *mRNA hybridisation showed no TNFR1 detection in the naive DRG. **(c) ***In situ *hybridisation for TNFR1 in the ipsilateral DRG 7 days after inflammation showed receptor expression in neuronal cells. *P < 0.05, **P < 0.01.

In order to investigate changes in TNF receptor expression in response to inflammation, we performed real-time RT-PCR and *in situ *mRNA hybridisation using gene-specific primers on DRG tissues taken from normal animals and at 3- and 7-day time points after induction of CFA arthritis. Real-time RT-PCR showed a modest expression of TNFR1 mRNA in naive tissues, with a threefold increase in TNFR1 mRNA in both the ipsilateral and contralateral DRG at 3 and 7 days after induction of inflammation (Fig. 2a). *In situ *hybridisation failed to detect TNFR1 mRNA in the naive rat (Fig. 2b) but identified TNFR1 mRNA in DRG cells at 3 and 7 days (Fig. 2c).

**Figure 3:**
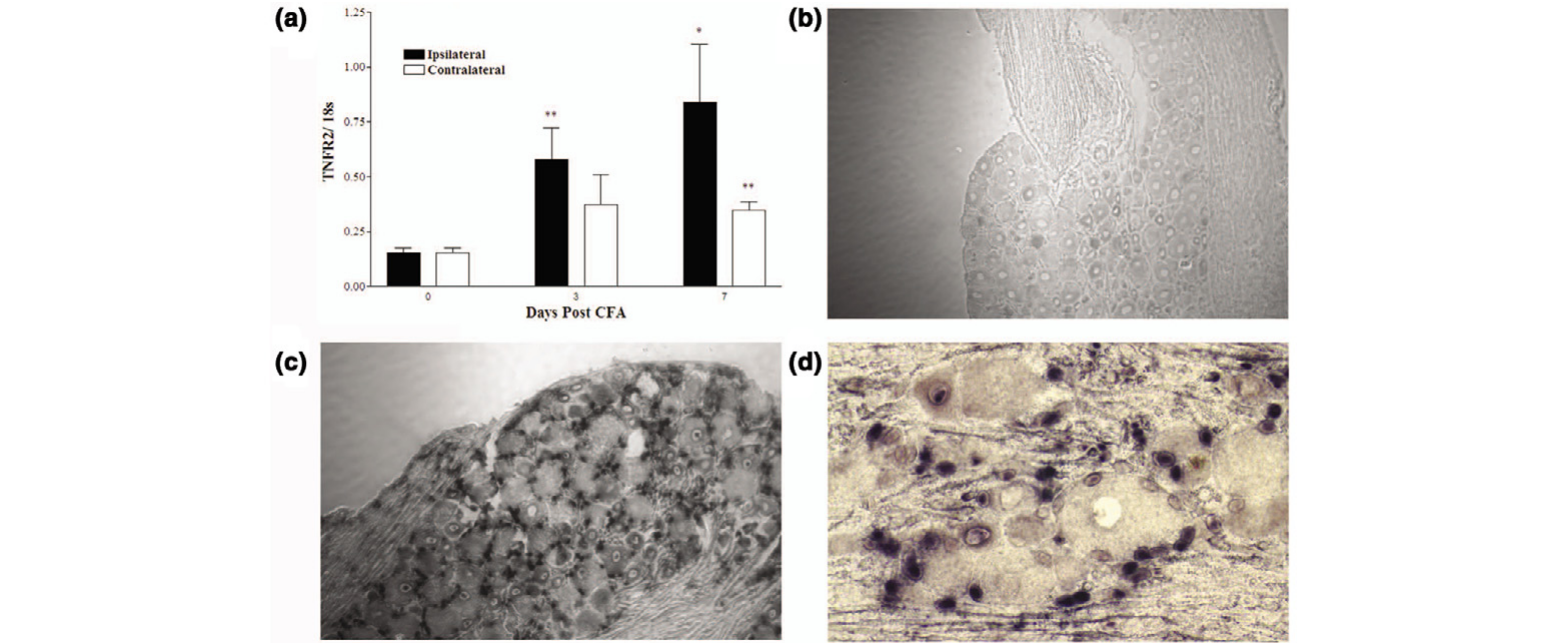
TNFR2 mRNA is increased following inflammation in the dorsal root ganglion (DRG). **(a) **Real-time RT-PCR showed an increase in mRNA of tumour necrosis factor receptor type 2 (TNFR2) (expressed as the ratio of TNFR2 mRNA to 18S mRNA) in the DRG ipsilateral to inflammation (*n *= 4), reaching five times its original level by 7 days. Contralateral TNFR2 levels increased significantly 7 days after inflammation. **(b) ***In situ *mRNA hybridisation in the naive DRG detected no TNFR2 mRNA. **(c) ***In situ *hybridisation detected TNFR2 mRNA in small cells of the DRG surrounding neuronal somata in the ipsilateral DRG 7 days after inflammation. **(d) **High-power magnification of TNFR2-labelled cells in the DRG, showing a perisomal distribution. *P < 0.05, **P < 0.01. CFA, complete Freund's adjuvant.

Expression of TNFR2 mRNA measured by real-time RT-PCR was increased after the induction of inflammation (Fig. 3a), rising to four times the initial level by 7 days after induction of inflammation. In contrast to TNFR1, TNFR2 mRNA was predominantly increased in the ipsilateral DRG. *In situ *hybridisation did not detect TNFR2 mRNA in the DRGs of naive animals (Fig. 3b) but identified ipsilateral expression of TNFR2 mRNA in small, non-neuronal cells 3 and 7 days after induction of inflammation (Fig. 3c,d).

### Development of antibody against TNFR2 by subtractive phage display

The cellular distribution of TNFR2 in DRG tissues remains controversial, and in the absence of a specific antibody to TNFR2 for immunohistochemical use, studies to date have mostly relied on identifying TNFR2 mRNA in DRG sections or ganglia extracts. In order to overcome this problem, we produced a specific anti-TNFR2 ScFv for use in immunohistochemical studies in the rat.

**Figure 4:**
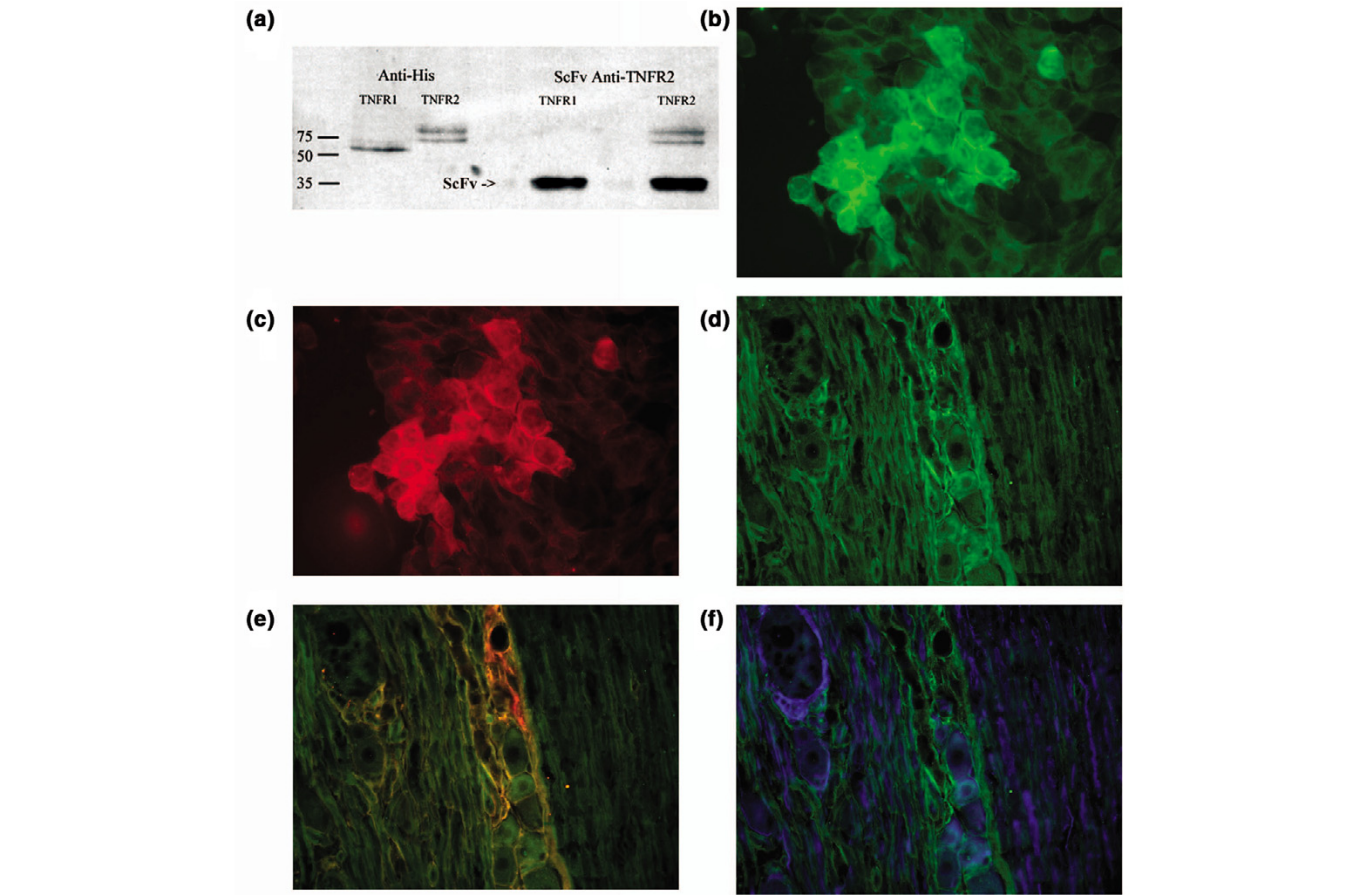
Monitoring expression of TNFR2 with anti-TNFR2 single-chain variable fragment (ScFv). **(a) **Immunoprecipitation of His-tagged tumour necrosis factor types 1 and 2 (TNFR1 and TNFR2) with anti-His antibody (left panel), and anti-TNFR2 single-chain variable fragment (ScFv) (right panel). The ScFv precipitated TNFR2, at ~75 KDa, but not TNFR1, at ~55 KDa, while the anti-His antibody precipitated both receptors, indicating specificity of the ScFv. Double immunohistochemistry against TNFR2-transfected 293T cells with anti-His antibody **(b)**, and ScFv plus anti-myc-CY-3 **(c) **indicates that the selected ScFv specifically binds toTNFR2. **(d) **Immunohistochemistry with anti-TNFR2 ScFv in the dorsal root ganglion 7 days after inflammation ipsilateral to injection. TNFR2 colocalisation was observed with the macrophage marker, ED1 **(e) **but not with glial fibrillary acidic protein (GFAP) **(f)**, indicating expression of TNFR2 by macrophages following inflammation.

TNFR1 and TNFR2 were cloned with a histidine tag and were then used to transfect 293T cells. Subsequently, phage display human antibody library was used to produce a ScFv against TNFR2 employing 293T cells transfected with TNFR2. Specificity of the ScFv was confirmed through ELISA (9.3-fold increased binding to TNFR2 relative to TNFR1). The ScFv was shown to immunoprecipitate TNFR2 but not TNFR1 (Fig. 4a). Specificity was also tested using immunohistochemistry against TNFR2-transfected 293T cells. An anti-His-FITC antibody was used to detect cells expressing the TNFR2 (Fig. 4b), and colocalisation was shown by double immunohistochemistry using the ScFv and an anti myc-CY-3 antibody (Fig. 4c).

### Characterisation of TNFR2-expressing DRG cells

The ScFv TNFR2 antibody described in the previous section was used in triple immunohistochemical studies to characterise TNFR2-expressing DRG cells in naive and arthritic rats. A monoclonal antibody against rat ED1 antigen was used to recognise cells of macrophage–monocyte lineage, and an antibody against glial fibrillary acidic protein (GFAP) was used to identify glial cells.

Seven days after CFA injection, TNFR2 protein was detected in small DRG cells (Fig. 4d). At all time points studied, TNFR2 colocalisation was observed exclusively with ED1-positive cells (Fig. 4e), and no colocalisation was observed with GFAP-positive cells (Fig. 4f).

**Figure 5:**
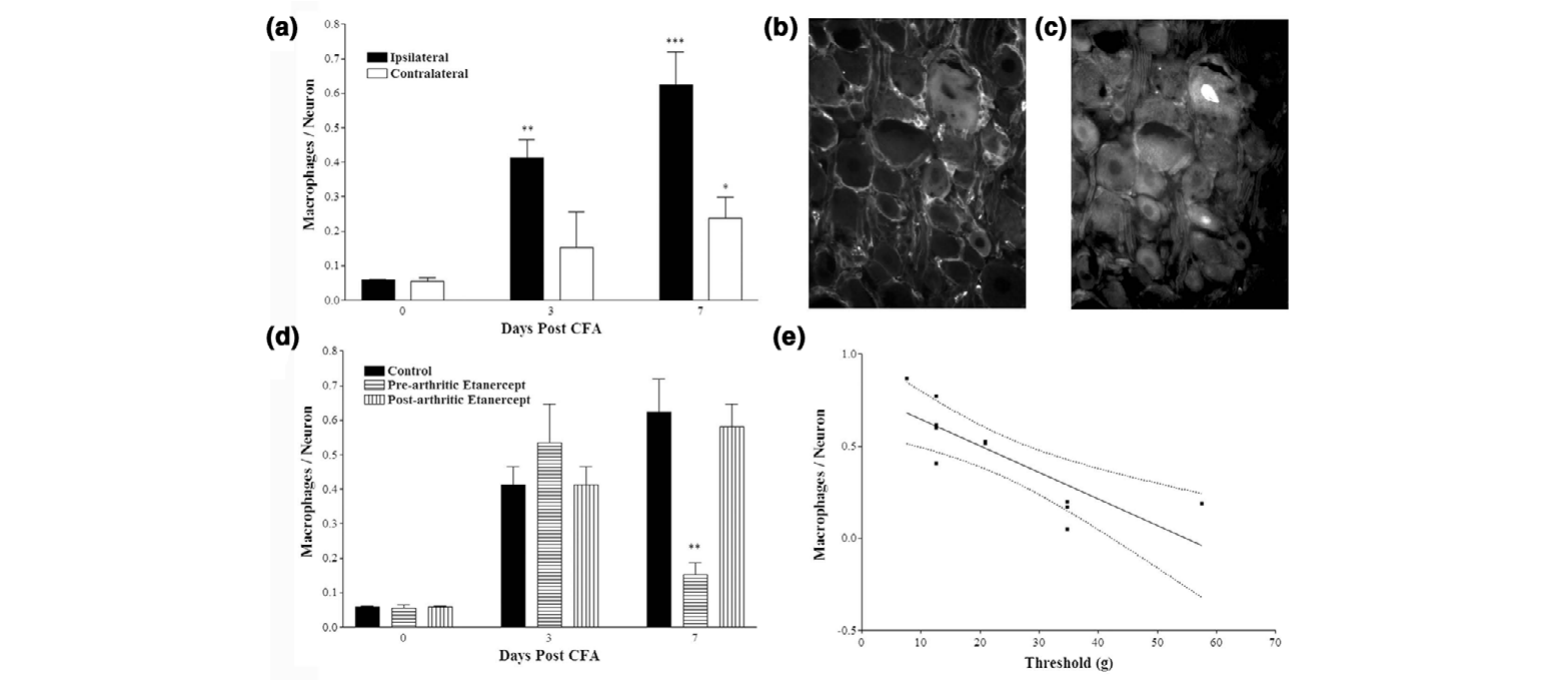
Prearthritic treatment with etanercept reduces postarthritic macrophage accumulation in the dorsal root ganglion (DRG). **(a) **Inflammation due to complete Freund's adjuvant (CFA) induced a 10-fold increase in perineuronal macrophages (*n *= 4) by 7 days after inflammation. Infiltration of macrophages was restricted to the ipsilateral side, except for 7 days after inflammation, when a threefold increase in perineuronal macrophages was observed in the contralateral DRG. Nerve damage was assessed through double immunohistochemistry using the macrophage marker, ED1 **(b) **and a neuropathic marker, ATF-3 (activating transcription factor-3) **(c)**. A maximum of two ATF-3-positive cells was detected per DRG, indicating that little nerve damage occurs in this model. **(d) **Prearthritic treatment with etanercept reduced macrophage numbers in the ipsilateral DRG by 75% 7 days after inflammation in comparison with controls treated with denatured etanercept (*n *= 4). No differences in macrophage numbers were detected with etanercept treatment commencing 3 days after inflammation induction. **(e) **A significant correlation between macrophage numbers in the DRG and mechanical hyperalgesia was observed 7 days after inflammation (*n *= 12). All treatment groups and controls were plotted. *R*^2 ^= 0.667, *P *< 0.01. *P < 0.05,**P < 0.01, ***P < 0.005.

Modest numbers of ED1-positive cells were observed in the naive rat DRG. Significantly, there were 7- and 10-fold increases in the ratio of ED1-positive cells to neuronal cells at, respectively, 3 and 7 days after CFA injection in the ipsilateral DRG (Fig. 5a). A less marked increase in ED1-positive cell numbers was observed in the contralateral DRG, reaching statistical significance only at 7 days.

In order to assess for possible neuronal injury in the CFA model, we used double immunohistochemistry with ED1 (Fig. 5b) and activating transcription factor-3 (ATF-3), a marker of nerve damage (Fig. 5c.) At all assessed time points, little or no ATF-3 expression was observed, with a maximum of two ATF-3-positive cells per DRG section.

### Effects of etanercept on ED1-positive cell numbers in rat DRG

Administration of etanercept before onset of CFA-induced inflammation produced a 75% reduction in ED1-positive cell numbers in the ipsilateral DRG in comparison with controls at 7 days (Fig. 5d). In contrast, treatment with etanercept after the onset of inflammation had no effect on ED1-positive cell numbers at the same time point (Fig. 5d). A highly significant correlation between numbers of ED1-positive cells in the DRG and development of mechanical hyperalgesia was observed 7 days after inflammation (R^2 ^= 0.677, *P *< 0.01.)

## Discussion

This study has provided several novel findings. One is that etanercept exerts a differential effect on the thermal and mechanical hyperalgesia associated with rat CFA-induced arthritis. Another is that experimental joint inflammation is associated with increased TNF-receptor expression on DRG cells that is maintained through the time course of the model. We also found that DRG TNFR2 expression occurs exclusively on non-neuronal cells of the macrophage–monocyte lineage, with cell numbers increasing in a TNF-dependent fashion during CFA arthritis. And, finally, we found a strong correlation between numbers of macrophage–monocyte cells and development of mechanical hyperalgesia in CFA arthritis.

CFA-induced arthritis has been used extensively in studies of behavioural pain responses [29,30]. Three hours after induction of inflammation, TNF is significantly up-regulated in local tissues, with the rise being maintained for a minimum of 5 days [3]. Consistent with a nociceptive role for TNF in this and other experimental models, antisera to TNF have been reported to transiently reduce thermal and mechanical hyperalgesia early in the course of CFA arthritis (within 24 hours) as well as after intraplantar injection of carageenan and also lipopolysaccharide, [31]. The effect of anti-TNF treatment at later time points has not previously been reported; however, prior inflammation (of rat knee) has been shown to be associated with an increased sensitivity to TNF [32], suggesting the presence of a dynamic, time-dependent process.

Etanercept had little or no effect on pain-related behaviour in naive animals. This observation accords with the observation that TNF administration into noninflamed tissues induces only modest hyperalgesia [33]. It is also consistent with the present finding of relatively low-grade TNF receptor expression in both neuronal and non-neuronal DRG cells taken from animals without inflammation.

In this study, the hyperalgesic response to thermal stimuli was reduced by etanercept, irrespective of whether it was administered before or after the onset of inflammation. In contrast, etanercept reduced mechanical hyperalgesia only when administered before the onset of inflammation. These results imply that although TNF plays a role in both the thermal and the mechanical hyperalgesia that accompanies inflammation, different mechanisms may be operating.

After induction of CFA arthritis, expression of both TNFR1 and TNFR2 within the DRG tissues was markedly increased. The observation that neuronal expression of neuronal TNFR1 increased with time confirms and extends previous observations made in neuropathic models [15,13]. It also accords with the time-dependent effects of etanercept, which was effective at reducing thermal hyperalgesia at 7 days but not at earlier time points of the experimental model, when TNFR1 expression was less apparent. This evolving pattern mirrors that observed after spinal ligation, which is associated with increasing sensitivity of both injured and noninjured neurons to TNF [34].

In contrast to TNFR1, we found no evidence for neuronal expression of TNFR2. Previously, TNFR2 has variously been reported either as being expressed on neuronal cells [11,12] or as not being expressed [13]. In our study, we used a highly specific antibody to TNFR2 protein, and although low-grade neuronal TNFR2 expression may have been below the detection threshold for the immunocytochemical technique used, it seems unlikely that functionally important expression was present.

TNFR2 was, however, shown to be expressed by non-neuronal cells of macrophage–monocyte lineage. The expression of TNFR2 increased significantly during the time course of CFA arthritis as a result of increased numbers of ED1-positive cells in the DRG following inflammation. To our knowledge, macrophage invasion of the DRG has previously been reported only in association with nerve injury [28]. In neuropathic models, ATF-3, a member of the ATF/CREB family, is up-regulated in damaged neurons [35]. Although its role after nerve damage is not known, it is regarded as a unique neurochemical marker of nerve injury. Little or no ATF-3 DRG expression was observed at any time point during CFA-induced inflammation; this observation provides strong evidence that there is not significant nerve injury in the CFA model.

A unique finding in the present study is that there was a highly significant correlation between macrophage numbers and the mechanical pain threshold 7 days after the onset of inflammation (Fig. 5e). Pretreatment with etanercept virtually abolished CFA-related macrophage infiltration, while substantially reducing the development of mechanical hyperalgesia. Paradoxically, treatment after the onset of inflammation had no effect on macrophage numbers and did not ameliorate mechanical hyperalgesia.

The antihyperalgesic properties of etanercept observed in the CFA model show striking similarities to those observed in chronic constriction injury of peripheral nerves [16]. Etanercept treatment that is started before the nerve is injured reduces subsequent mechanical hyperalgesia dramatically, while treatment started after injury has little effect. In addition, both prophylactic and postconstriction treatment with etanercept in the chronic-constriction-injury model reduces thermal hyperalgesia only at later time points. The magnitude of the infiltration of macrophages into the DRG after induction of CFA arthritis was of a similar degree to that observed following axotomy [28], leading us to question both the mechanism leading to the infiltration and the resulting functional role that DRG macrophages might play in hyperalgesic responses in models of inflammatory disease.

Traditionally, macrophage infiltration into the DRG has been thought to play a key role in degeneration and repair of the damaged nervous system [36]. However, the apparent absence of nerve damage in the CFA arthritis model leads to speculation that DRG macrophage infiltration may play additional roles. Depletion of macrophages during neuropathic conditions reduces hyperalgesia [37]. A study comparing macrophage infiltration into the DRG in neuropathic models [38] found low-level macrophage infiltration in models with extensive nerve damage, whereas macrophage numbers were highest in models with the greatest mechanical hyperalgesia. The conclusion was that DRG macrophages played a more important role in the genesis of hyperalgesia than in repair of neuronal damage. It therefore seems likely that DRG macrophages play a role in hyperalgesic states associated both with neuronal injury and with conditions associated with chronic inflammation.

## Conclusion

This study has shown a differential effect of anti-TNF therapy on thermal and mechanical inflammatory hyperalgesia. Inflammation is associated with increased TNFR1 expression by neuronal cells, potentially leading to direct activation of these cells by TNF under inflammatory conditions. Inflammation was associated with a TNFR2-dependent accumulation of macrophages into the DRG. Macrophage infiltration into the DRG has previously only been reported following nerve injury and has been linked to a role in tissue damage and repair. The correlation between DRG macrophage numbers and mechanical hyperalgesia suggests a much broader role for these cells in the maintenance of chronic pain states. The analgesic efficacy of anti-TNF therapies in human arthropathies attests to the importance of TNF in the pathogenesis of chronic arthritic pain. These results indicate that TNF makes a more direct contribution to chronic inflammatory pain than has hitherto been assumed.

**Figure 6:**
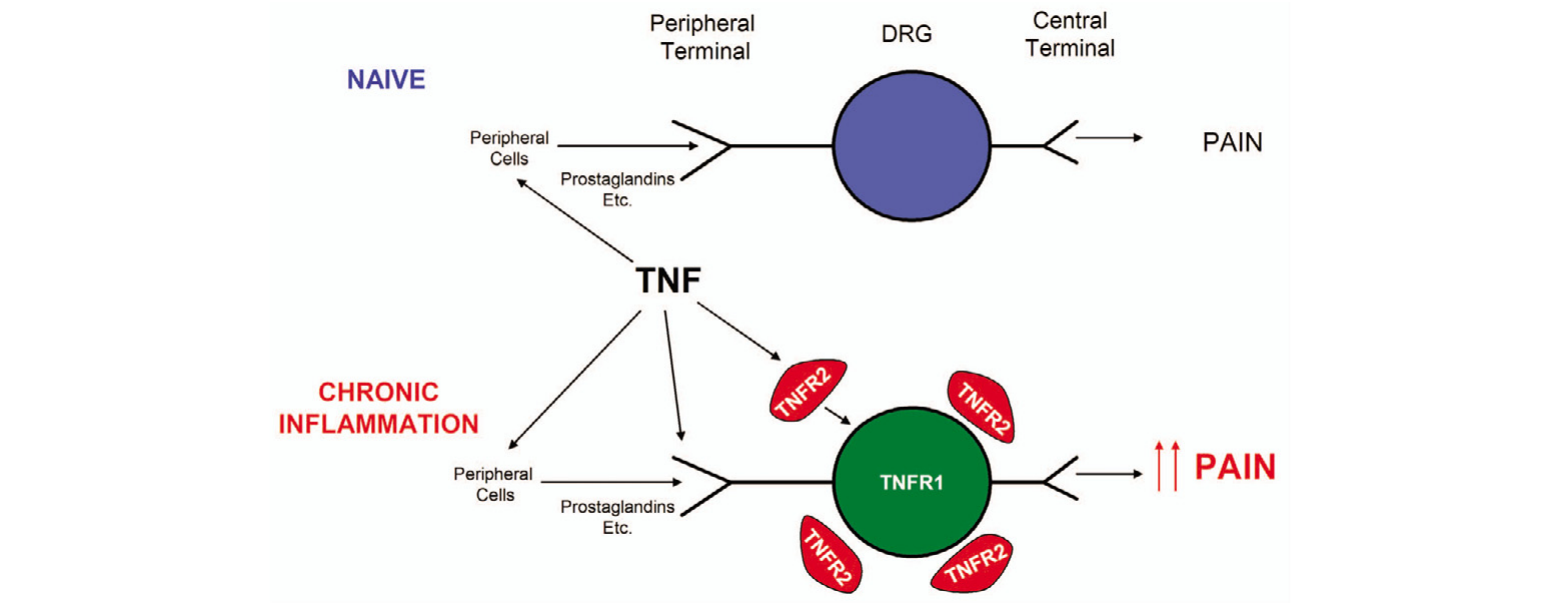
Actions of tumour necrosis factor (TNF) on hyperalgesia in health and in chronic inflammation. In the naive dorsal root ganglion (DRG), TNFα acts on peripheral cells to induce a proinflammatory cascade resulting in the release of mediators, such as prostaglandins, that activate nociceptive neurons, resulting in pain. After chronic inflammation, tumour necrosis factor receptor type 1(TNFR1) is up-regulated on DRG neurons, while TNFR2 is expressed by infiltrating macrophages. TNF can directly modulate neuronal function and act on peripheral cells and DRG macrophages to induce inflammatory mediators that can modulate neuronal function. This results in exaggerated pain.

In summary, present evidence suggests that under noninflammatory conditions, TNF-α acts on peripheral cells to induce a proinflammatory cascade resulting in the release of additional mediators to sensitise and activate nociceptive neurons. The present study has shown that chronic inflammation is associated with up-regulation of TNFR1 on DRG neurons, thereby providing an opportunity for direct interaction between TNF and sensory neurons. This study has also shown the presence of TNFR2-expressing infiltrating macrophages, providing a second pathway by which TNF can modulate neuronal function in inflammatory as well as neuropathic conditions (see Fig. 6).

## Abbreviations

ATF-3 = activating transcription factor-3; CFA = complete Freund's adjuvant; DRG = dorsal root ganglion; ELISA = enzyme-linked immunosorbent assay; FITC, fluorescein isothiocyanate; GFAP = glial fibrillary acidic protein; L4/L5 = fourth/fifth lumbar vertebrae; PBS = phosphate-buffered saline; ScFv = single-chain variable fragment; TNF = tumour necrosis factor; TNFR(1/2) = tumour necrosis factor receptor (type 1/2).

## Competing interests

The author(s) declare that they have no competing interests.

## Authors' contributions

JJI carried out the study and analysed the data. JJI, BLK, YC, and SPH designed the study. AN contributed to the phage display experimental design. YC contributed to the cloning and expression of the TNFRs and riboprobes. DML assisted with the real-time RT-PCR experiments. All authors read and approved the final manuscript.
